# Development of a Mobile-Based Personal Health Record for Pediatric Attention-Deficit/Hyperactivity Disorder Management: Protocol for a Study Based on Action Research Design

**DOI:** 10.2196/60216

**Published:** 2025-04-10

**Authors:** Dian Budi Santoso, Martina Sinta Kristanti, Dian Kesumapramudya Nurputra, Retno Sutomo

**Affiliations:** 1 Department of Health Information and Services Vocational College Universitas Gadjah Mada Special Region of Yogyakarta Indonesia; 2 Doctoral Program in Medical and Health Sciences Faculty of Medicine, Public Health, and Nursing Universitas Gadjah Mada Special Region of Yogyakarta Indonesia; 3 Department of Basic and Emergency Nursing Faculty of Medicine, Public Health, and Nursing Universitas Gajah Mada Special Region of Yogyakarta Indonesia; 4 Department of Child Health Faculty of Medicine, Public Health, and Nursing Universitas Gajah Mada Special Region of Yogyakarta Indonesia

**Keywords:** ADHD, attention deficit and hyperactivity, mobile app, personal health records, action research, pediatric

## Abstract

**Background:**

Attention-deficit/hyperactivity disorder (ADHD) is one of the most widespread neurobehavioral problems during childhood. A child’s personal health record (PHR) plays an important role in the controlled routine monitoring of ADHD symptom improvement. Along with the advantages, the convenience offered by mobile technology, and the ubiquity of smartphones in contemporary society, there is a compelling need for PHR to be available in the form of a mobile app.

**Objective:**

This study aims to identify stakeholder needs, followed by designing, developing, testing, and evaluating a mobile-based PHR in the context of pediatric ADHD management.

**Methods:**

This study will adopt an action research design structured into 4 stages: diagnosing, planning, taking, and evaluating action. Stakeholders, including parents, pediatricians, occupational therapists, clinical psychologists, and teachers, will participate actively. In stage 1, stakeholder requirements for the mobile-based PHR will be explored through in-depth interviews, focus group discussions (FGDs), and document reviews. Thematic analysis will be used to identify key needs and challenges. In stage 2, a systematic literature review will be conducted to enhance user requirements analysis by synthesizing insights from existing mobile apps for pediatric ADHD management. A mobile-based PHR prototype will be designed and developed based on user requirements enhanced with systematic review results. In stage 3, the prototype will undergo a 6-week trial with participants to evaluate its functionality and address any identified issues. In stage 4, both quantitative and qualitative methods will be used to assess the app’s usability and quality. The System Usability Scale (SUS) and the User Version of the Mobile App Rating Scale (uMARS) will be used for quantitative evaluation, while interviews and FGDs will be conducted for qualitative evaluation.

**Results:**

This study commenced in October 2024. As of December 2024, 13 participants (n=5, 38.5%, parents; n=2, 15.4%, pediatricians; n=2, 15.4%, occupational therapists; n=2, 15.4%, clinical psychologists; and n=2, 15.4%, teachers) have been enrolled, meeting the minimum participant requirements for stage 1. Stage 1 was completed at the end of 2024, with stage 2 expected to be completed by September 2025, stage 3 by December 2025, and stage 4 by February 2026. The findings from each stage will inform iterative refinements to the mobile-based PHR. The final results, including usability and quality assessments, are anticipated for publication by the middle of 2026.

**Conclusions:**

This study protocol outlines a pivotal initiative to enhance the management of pediatric ADHD. By using an action research methodology and actively engaging stakeholders, the study aims to contribute significantly to the field. The iterative cycles of the research seek to develop a mobile-based PHR that is not only user friendly but also effective and uniquely attuned to the diverse needs of those involved in pediatric ADHD care.

**International Registered Report Identifier (IRRID):**

PRR1-10.2196/60216

## Introduction

### Background

Attention-deficit/hyperactivity disorder (ADHD) is one of the most widespread neurobehavioral problems during childhood, with a worldwide prevalence of 5.29% [1,2]. The manifestation of ADHD occurs in 5.9% of youth and 2.5% of adults [3]. In the United States, an estimated 11%, equivalent to approximately 6.4 million children and adolescents aged 4-17 years, have received a diagnosis of ADHD [4]. Characterized by persistent patterns of inattention, hyperactivity, and impulsivity, ADHD manifests in various ways, affecting all aspects of a child’s life [5,6]. The impact of ADHD extends beyond the immediate challenges of managing symptoms; it often disrupts academic performance, hinders peer relationships, and can contribute to emotional and behavioral difficulties [7-9]. Children with ADHD may face an increased risk of academic underachievement, low self-esteem, and a higher likelihood of engaging in risky behaviors [10-12]. Moreover, the condition places considerable stress on families and educators as they navigate the complexities of providing optimal support [13,14]. Recognizing the widespread prevalence and multifaceted impact of ADHD is crucial for developing targeted interventions and support systems that address the unique needs of affected children and related stakeholders.

Addressing the diverse needs of stakeholders in managing ADHD takes on added significance when considering the integration of personal health records (PHRs) into the care framework [15]. The PHR can serve as a central repository for health-related information, offering a comprehensive view of a child’s medical history, treatment plans, symptoms improvement, and daily challenges associated with ADHD [16]. Within the context of pediatric ADHD management, routine and controlled monitoring of symptom improvement plays a pivotal role, and the PHR become instrumental in this process [17]. The PHR provides a structured platform for caregivers and related stakeholders to systematically monitor and analyze the child’s response to therapeutic interventions [18]. By documenting the symptoms’ progression, medication adherence, and the impact of various strategies, the PHR offers valuable insights that facilitate evidence-based decision-making [19]. Moreover, the ability to monitor symptoms in a controlled and consistent manner allows for a more nuanced and tailored approach to therapy adjustments, ensuring that interventions align closely with the child’s unique needs [20]. The incorporation of the PHR into pediatric ADHD management not only enhances the accessibility of critical health information but also empowers stakeholders with a comprehensive tool for informed decision-making and the continual refinement of therapeutic strategies.

Although the idea of a PHR has historical roots in paper-based systems, health information technology now enables individuals to electronically store and manage their health information, granting them convenient access, whenever necessary [21]. In addition, with the ubiquity of smartphones in contemporary society, there is a compelling need for the PHR to be available in the form of a mobile app [22,23]. Furthermore, technological interventions for the assessment, monitoring, and treatment of neurodevelopmental disorders, including ADHD, are increasingly focused on mobile apps [24]. However, few apps related to ADHD provide information about their development processes, and none include evidence supporting their effectiveness [25].

The urgency to develop a mobile-based PHR specifically tailored for pediatric ADHD management stems from the growing demand for facilitating information sharing between stakeholders to promote the adoption of evidence-based practices in the management of children with ADHD [26]. The fragmentation of health care services, coupled with the dynamic nature of ADHD symptoms, necessitates a technological solution that can bridge communication gaps and empower those involved in the child’s care [26,27].

Recognizing the imperative for a user-centric approach, the adoption of an action research methodology becomes paramount in the development of a mobile-based app [28-30]. Action research, designed to enhance the engagement of potential mobile app users in the entire research process, can foster awareness within the pediatric ADHD management stakeholders’ context of how they experience and interact with technology [31,32]. This inclusive approach ensures that the resulting mobile app is not only technologically sound but also rooted in the practical experiences, preferences, and needs of those directly engaged in the daily challenges of pediatric ADHD management. Through iterative cycles, the action research methodology contributes to the development of an effective, user-friendly, and contextually relevant mobile-based PHR, ultimately advancing the landscape of pediatric ADHD management via the active participation of the stakeholders.

### Objectives

The objectives of this study are to first identify the needs of stakeholders concerning the development of a mobile-based PHR for pediatric ADHD management. Based on the identified needs, a customized mobile app will be developed collaboratively, with continuous input from the stakeholders to ensure the app addresses those needs effectively. Finally, the usability and quality of the mobile app will be rigorously tested and assessed, ensuring it aligns with the preferences and expectations of the stakeholders and functions as an effective tool for managing pediatric ADHD.

## Methods

### Study Design

This study will use an action research methodology in the form of a cycle with 4 stages (diagnosing action, planning action, taking action, and evaluating action), integrating both qualitative and quantitative designs [33,34]. The term “action research” in this paper refers to the same definition as “participatory action research,” “participatory research,” “interactive research,” “collaborative inquiry/research,” and “engaged scholarship” [35]. The action research design centers on doing “with” rather than doing “for” relevant stakeholders [36]. In this context, the participation of the relevant stakeholders becomes key to the implementation of action research.

**Figure 1:**
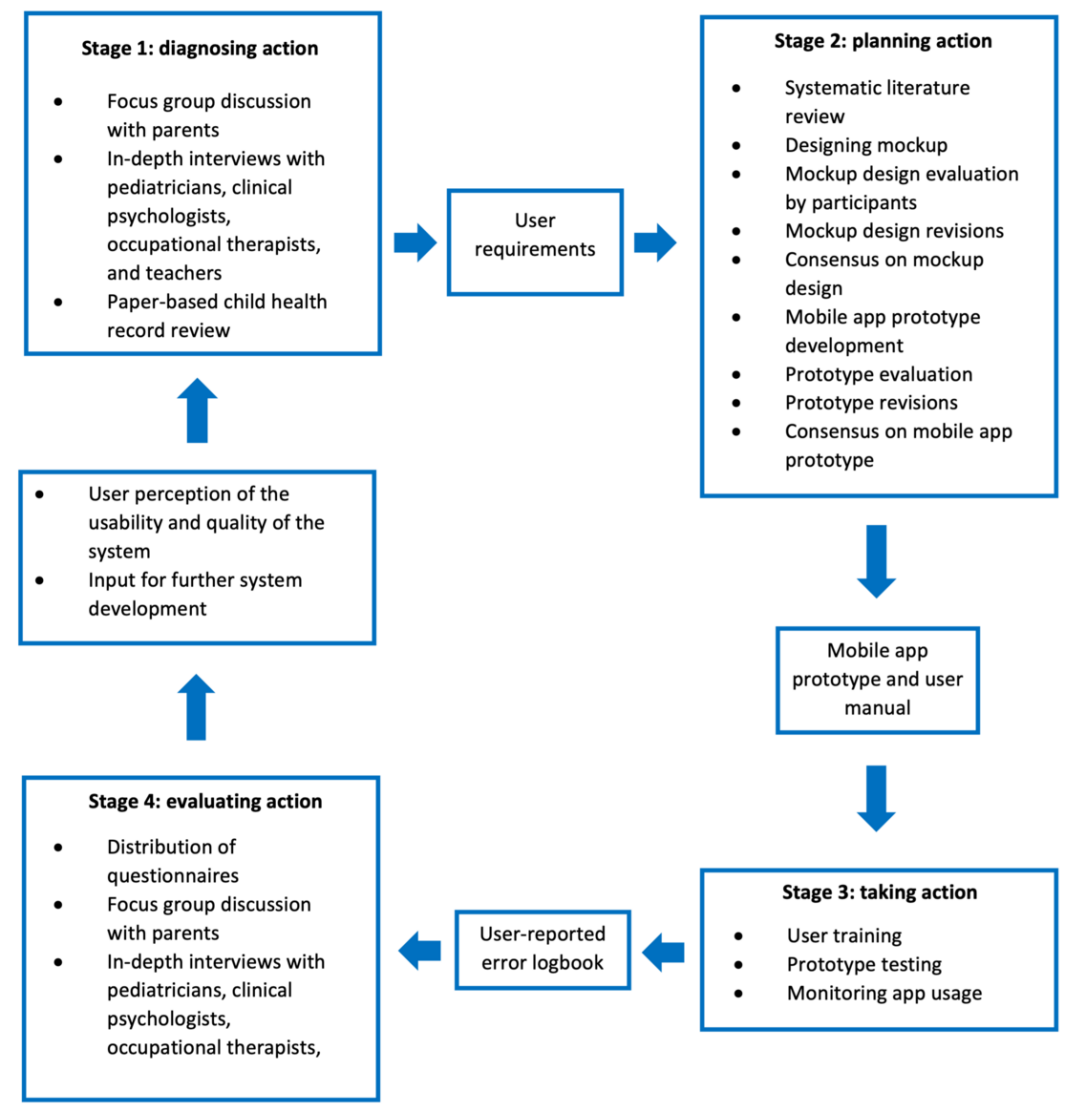
Stages of action research.

Each stage in action research consists of an iterative process (plan, act, observe, reflect), involving the stakeholders at each step [37]. The stages of action research in this study can be succinctly observed in Figure 1.

Table 1 outlines the specific steps in each stage of action research, the measures used to track progress, and the timeline for each activity. Activities at each stage will be carried out, and their progress will be systematically monitored based on the table.

**Table 1:** Detailed steps and measures in each stage of the study.

Stage and steps			Measure(s)		Timeline
**Stage 1: diagnosing action**					
	Conduct user requirement analysis through FGDs^a^ with parents and interviews with pediatricians, occupational therapists, clinical psychologists, and teachers.	Number of participants, identified user needs		Months 1 and 2	
	Review paper-based documents managed by parents and related stakeholders.	Completeness of documentation analysis		Months 1 and 2	
**Stage 2: planning action**					
	Conduct a systematic literature review on the mobile-based app used for pediatric ADHD^b^ management.	Number of reviewed studies, extracted key findings		Months 3-5	
	Design a mobile-based PHR^c^ mockup.	Initial mockup completion		Month 6	
	Review and revise the mockup design based on participant feedback until consensus is reached.	Number of revisions, participant consensus		Month 7	
	Develop a prototype based on the final mockup design.	Initial prototype completion		Months 7-10	
	Review and revise the prototype based on participant feedback until consensus is reached.	Number of revisions, participant consensus		Months 11 and 12	
**Stage 3: taking action**					
	Conduct training and mentoring for participants on using the mobile-based PHR.	Participant attendance, knowledge gained		Month 13	
	Facilitate prototype trial usage among participants.	Number of users engaged		Months 14 and 15	
	Monitor app usage to identify challenges or technical issues.	Number of reported issues		Months 14 and 15	
**Stage 4: evaluating action**					
	Assess system usability using the SUS^d^ questionnaire.	SUS score (range 0-100)		Month 15	
	Evaluate app quality using the uMARS^e^ questionnaire.	Mean (SD) of uMARS scores		Month 15	
	Collect qualitative feedback via FGDs and interviews.	Identified feedback		Months 15 and 16	
	Summarize findings and provide recommendations for future development.	Final report completion		Months 17 and 18	

^a^FGD: focus group discussion.

^b^ADHD: attention-deficit/hyperactivity disorder.

^c^PHR: personal health record.

^d^SUS: System Usability Scale.

^e^uMARS: User Version of the Mobile App Rating Scale.

### Study Setting

This research will be conducted at Dr Sardjito Hospital, Special Region of Yogyakarta, Indonesia. This hospital was chosen as the research location because it is an educational and referral hospital with a pediatric health clinic specializing in social pediatrics and neurology, which is commonly referred to by patients with ADHD. As a tertiary hospital, it receives a significant number of pediatric patients with ADHD referred there each year. The hospital’s comprehensive health care facilities and expertise in pediatric care, particularly in social pediatrics and neurology, make it an optimal environment for conducting this research.

This research will also be conducted in schools where children with ADHD receive formal education in Indonesia. These children are aged between 4 and 12 years [38]. Teachers who work with children with ADHD, including preschool and elementary school teachers, will be actively involved in the study. It is widely recognized that communication and active involvement of parents, health care providers, and teachers are essential for managing pediatric ADHD [39].

### Stage 1: Diagnosing Action

#### Objective

The objective of stage 1 (diagnosing action) is to systematically identify and understand the diverse needs of stakeholders in relation to the development of a mobile-based PHR. This initial phase will be dedicated to diagnosing and comprehending the requirements of key stakeholders involved, including parents, pediatricians, occupational therapists, clinical psychologists, and teachers of children with ADHD. To develop high-quality apps, it is imperative to build the development process upon a comprehensive understanding of user requirements, derived from a methodology that actively involves users in the process [40]. Through a meticulous process involving document review, focus group discussions (FGDs), and in-depth interviews, the stage will seek to uncover issues related to the recording of ADHD symptom development by parents and the PHR’s role as a communication medium among parents, teachers, and health care professionals. The outcomes of this stage will include a comprehensive analysis of stakeholder needs, outlining specific requirements for the development of a mobile-based PHR.

#### Output

The output of this stage will be a document containing specific requirements for the development of a mobile-based PHR. These requirements include data needs, user groups and permissions, app features, functionalities, and expected technology specifications.

#### Participants

Stakeholders involved in the management of children with ADHD, including parents, pediatricians, occupational therapists, clinical psychologists, and teachers, will participate in this study [41,42]. Selection criteria for research participants are detailed in Table 2.

Involvement of parents or caregivers and teachers is related to the assessment of ADHD that requires evidence directly obtained from them regarding the core symptoms of ADHD, the duration of symptoms, the degree of functional impairment, and associated conditions [43].

We estimate that our study will need around 5 parents, 2 pediatricians, 2 occupational therapists, 2 clinical psychologists, and 2 teachers. This is the minimum number of participants; additional participants may be included during data collection until data saturation is achieved [44].

**Table 2:** Participant selection criteria.

Participants	Criteria
Parents	Parents of children diagnosed with ADHD^a^ (aged 4-12 years) who have received behavioral therapy and medication, with at least 1 year of experience in taking care of children with ADHD Experienced in using mobile health apps Own and actively use an Android- or iOS-based mobile phone with an internet connection
Pediatricians	Pediatricians managing patients with ADHD whose parents are participating in the research, with a minimum of 2 years of experience in managing children with ADHD Currently pursuing or have completed subspecialization in developmental pediatrics–social pediatrics or child neurology Experienced in using electronic medical record systems or telemedicine or both
Occupational therapists and clinical psychologists	Occupational therapists and clinical psychologists managing patients with ADHD whose parents are participating in the research, with a minimum of 2 years of experience in managing children with ADHD Experienced in using electronic medical record systems or telemedicine or both
Teachers	School teachers teaching children with ADHD whose parents are participating in the research, with a minimum of 2 years of experience in teaching children with ADHD Own and actively use an Android- or iOS-based mobile phone with an internet connection

^a^ADHD: attention-deficit/hyperactivity disorder.

#### Data Collection

Data will be collected through a systematic approach, including FGDs with parents and in-depth interviews with pediatricians, occupational therapists, clinical psychologists, and teachers. The total number of interviews will be adjusted based on the number of participants involved. Participants will be selected using a purposive sampling method based on predetermined inclusion criteria to ensure representation of key stakeholders actively involved in managing pediatric ADHD. Comprehensive FGD and interview guides have been developed in Indonesian to facilitate semistructured sessions. The inquiry framework acknowledges the dynamic nature of discussions and interviews, allowing flexibility in question sequencing, particularly regarding follow-up inquiries, which may undergo alterations during the research [45]. The guides are designed to explore specific aspects described in Table 3.

The data collection process will follow a systematic procedure. Participants will be recruited through referrals from pediatricians specializing in ADHD to ensure a diverse and representative sample. In-depth interviews and FGDs will be conducted in a private and comfortable environment to encourage open and honest responses. Each session will begin with an introduction explaining the purpose of the study and obtaining consent for audio recording.

All interviews and FGDs will be audio-recorded with the participants’ consent, and field notes will be taken to capture contextual details, nonverbal cues, and relevant observations during the sessions. Each in-depth interview session will be conducted for approximately 30-60 minutes, while each FGD session is expected to last from 60 to 120 minutes [46,47]. The semistructured nature of the interviews and FGDs will allow flexibility for follow-up questions and enable a deeper exploration of emerging themes during the discussions.

**Table 3:** Key aspects explored in stakeholder interviews and FGDs^a^.

Aspect	Focus of exploration
Stakeholder needs	Essential features required for a mobile-based PHR^b^ Information that needs to be recorded, monitored, and accessed (eg, symptom tracking, medication schedules, therapy progress)
Usability preferences	Design expectations (eg, user-friendliness, interface layout) Accessibility concerns, including navigation simplicity for parents and professionals
Data accessibility and security	Preferences for access control (eg, who can input and view data) Concerns about privacy and confidentiality of ADHD^c^-related information
Roles of stakeholders	How each stakeholder envisions contributing to and using the PHR Collaboration needs between parents, pediatricians, occupational therapists, clinical psychologists, and teachers in managing pediatric ADHD

^a^FGD: focus group discussion.

^b^PHR: personal health record.

^c^ADHD: attention-deficit/hyperactivity disorder.

As a methodological triangulation measure, paper-based child health records managed by parents will be reviewed to validate the essential data needs in the PHR [48]. Parents will be encouraged to present their manually recorded data, which reflects their needs in managing the care of children with ADHD. This will facilitate researchers in scrutinizing and documenting the various data types encapsulated within these records.

#### Data Analysis

Qualitative data from FGDs and in-depth interviews will be analyzed using thematic analysis following the 6-phase approach [49,50]: (1) familiarization with the data, where researchers immerse themselves in the data by reading and re-reading transcripts; (2) generating initial codes, involving systematic coding of significant features of the data across the entire dataset; (3) searching for themes, where codes are organized into potential themes; (4) reviewing themes to refine and ensure coherence and distinction between them; (5) defining and naming themes, focusing on capturing the essence of each theme; and (6) producing a report that involves a narrative that integrates the themes with supporting evidence from the data [51]. To address methodological rigor, aspects of reflexivity, confirmability, dependability, credibility, and transferability will be considered throughout the analysis process. Reflexivity will be addressed by creating fieldnote reflections, where researchers will document critical interpersonal dynamics affecting participants and their data, record and reflect on decisions made, and highlight moments of analytic insight to enhance transparency and self-awareness throughout the research process [52,53]. Confirmability will be ensured through detailed documentation of the analytic process, and peer debriefing dependability will be supported by maintaining a clear audit trail of the analysis process [54]. To enhance credibility, member checking with selected participants will be conducted, and investigator triangulation will be implemented with 2 researchers collaboratively reviewing and comparing the codes and resolving any differences through discussion for the same text unit [55]. Transferability will be facilitated by providing detailed descriptions of the study context and participant characteristics in this protocol [54]. The qualitative data will be organized using Open Code software version 4.03, ensuring systematic handling and transparency of the coding process [56,57].

### Stage 2: Planning Action

#### Objective

Stage 2 (planning action) aims to design and develop a prototype of a mobile-based PHR that is agreed upon by all research participants. This stage will involve several key steps. First, a systematic literature review will be conducted on mobile apps used in pediatric ADHD management to enhance the user requirement analysis from stage 1 by thoroughly examining and synthesizing the existing literature. In the mobile app development process, understanding the efficient assessment of mobile apps, academic challenges, and other important aspects available in the existing literature is crucial [58]. The review will focus on identifying app features, purposes, target users, reported outcomes, and the measuring instruments used in previous studies. It will also address important considerations, such as the efficient assessment of mobile apps and academic challenges in the field. Next, a mockup design of the mobile-based PHR will be created based on the results of both the user requirement analysis and the systematic literature review. This mockup will be used to validate the user interface’s usability [59].

Finally, a mobile app prototype will be developed based on the collective agreement of research participants on the finalized mockup design. The prototype will be developed in collaboration with a team of developers who will be recruited as part of the technical team for this study. The prototype will be built using Flutter, a framework that allows for the development of apps on both Android and iOS platforms, which together dominate the mobile app market [60-62].

#### Output

The outcome of this stage will be a mobile-based PHR prototype for both Android and iOS platforms, which has gained consensus among all research participants and is ready for testing in the next stage. Furthermore, a user manual for the mobile app will be produced during this stage.

#### Participants

Participants in this stage will be stakeholders related to pediatric ADHD who have previously participated in stage 1. Participants in this stage will actively engage in the design and development process [63].

#### Data Collection

Data collection will involve several key steps. First, a systematic literature review will be conducted and documented following the PRISMA (Preferred Reporting Items for Systematic reviews and Meta-Analyses) 2020 guidelines [64]. To comprehensively explore the available literature on mobile apps for pediatric ADHD, a systematic search will be conducted across multiple databases, including PubMed, Scopus, Cochrane Library, and Google Scholar. The search strategy will involve a combination of MeSH (Medical Subject Headings) terms as controlled vocabulary terms and their relevant synonyms related to pediatric ADHD, mobile apps, and pediatric ADHD management stakeholders. MeSH terms used as keywords will include “parents,” “child,” “adolescent,” “family,” “caregivers,” “physicians,” “school teachers,” “pediatrics,” “mobile applications,” “smartphone,” “digital health” and “attention deficit disorder with hyperactivity.” The search strings will be tailored to the specific syntax and requirements of each database. The study/source selection process will follow a systematic and transparent approach. The process consists of 3 main steps. First, titles and abstracts of identified papers will be screened by 2 independent reviewers using predefined inclusion criteria. Any discrepancies will be resolved through discussion, and if necessary, a third reviewer will be consulted to facilitate consensus. Second, a full-text review of selected studies will assess their eligibility, with reasons for exclusion recorded and reported. Finally, relevant data will be extracted from the included studies. Subsequently, the data analysis will explore the apps’ features, purposes, reported outcomes, as well as variations in apps usage across different contexts and participant groups.

Information derived from the systematic literature review will be gathered and used to complement the results of the user requirement analysis, forming the foundation for the design and development of the mobile-based PHR. Next, the designed mockup will be presented to each participant for comprehensive feedback. Every discussion will be recorded, transcribed, and analyzed, with refinements made to the mockup design based on participant input. This iterative process will continue until a unanimous consensus is reached among all participants [65]. Finally, the approved mockup design will be transformed into a mobile-based app prototype. Similar to the mockup phase, the prototype will be discussed with each participant to gather further feedback The discussions will be recorded, transcribed, and analyzed, with subsequent revisions made to the prototype until a unanimous agreement is achieved among all participants.

#### Data Analysis

Data from the systematic literature review will be analyzed descriptively to map the user groups, as well as the apps’ features, purposes, and outcomes related to the implementation of mobile apps for pediatric ADHD. Additionally, participant feedback on the mockup design and prototype of the mobile-based PHR will be qualitatively analyzed, serving as a foundation for the continuous improvement and refinement of both the mockup design and the prototype.

### Stage 3: Taking Action

#### Objective

Stage 3 (taking action) will involve the trial of the mobile-based PHR. The objective of this stage is for participants to actively use the mobile app in their daily activities while monitoring children with ADHD. Throughout this stage, technical challenges faced by users during the trial period will be identified to facilitate app improvements and refinements.

#### Output

The output of this stage will be a logbook document detailing the errors or technical challenges reported by users during the trial period. This logbook will serve as a comprehensive record of user feedback, documenting encountered issues and discrepancies. It will function as a crucial control document for the debugging process and for implementing necessary system refinements [66]. By systematically logging user-reported errors, the logbook will become a valuable resource for the iterative improvement process, ensuring that the mobile app prototype is continually enhanced and optimized based on real-world user experiences.

#### Participants

Participants in this stage will include individuals who have previously participated in stages 1 and 2, along with newly recruited parents of children diagnosed with ADHD. The number of newly recruited parent participants is anticipated to range between 20 and 80 individuals [67]. Eligibility criteria for participation require owning a smartphone compatible with either the Android or the iOS platform and having reliable internet access.

#### Data Collection

In this stage, participants will be asked to use the mobile-based PHR for 6 weeks before proceeding to the next stage [68]. During the trial period, app usage will be monitored, and users will be asked to report any system errors or technical issues encountered through the reporting form available in the mobile-based PHR. The reported data will be recorded and compiled into an error logbook. This monitoring will allow user issues to be addressed promptly, enabling the technical team to provide targeted support, as needed. To ensure participants use the mobile-based PHR consistently during the 6-week period and can test its entirety, they will be provided with detailed instructions during user training, as well as regular reminders and support throughout the period.

#### Data Analysis

The collected data will be descriptively analyzed by grouping similar types of errors or technical issues. Subsequently, app improvements and refinements will be implemented based on the analysis. Users will be informed of the implemented improvements and refinements through notifications in the mobile-based PHR.

### Stage 4: Evaluating Action

#### Objective

Stage 4 (evaluating action) will focus on assessing the overall quality and usefulness of the mobile-based PHR from the user perspective. Unlike earlier stages, which focus on design, development, and refinement, this stage will primarily serve as a comprehensive evaluation of the outcomes of the previous stages. It will involve gathering feedback from participants on the effectiveness of the mobile-based PHR and identifying expectations for its further development.

#### Output

The output of stage 4 will be a comprehensive evaluation report that summarizes participants’ assessments regarding the usefulness and quality of the mobile-based PHR. Additionally, this stage aims to generate valuable feedback for future development.

#### Participants

**Figure 2:**
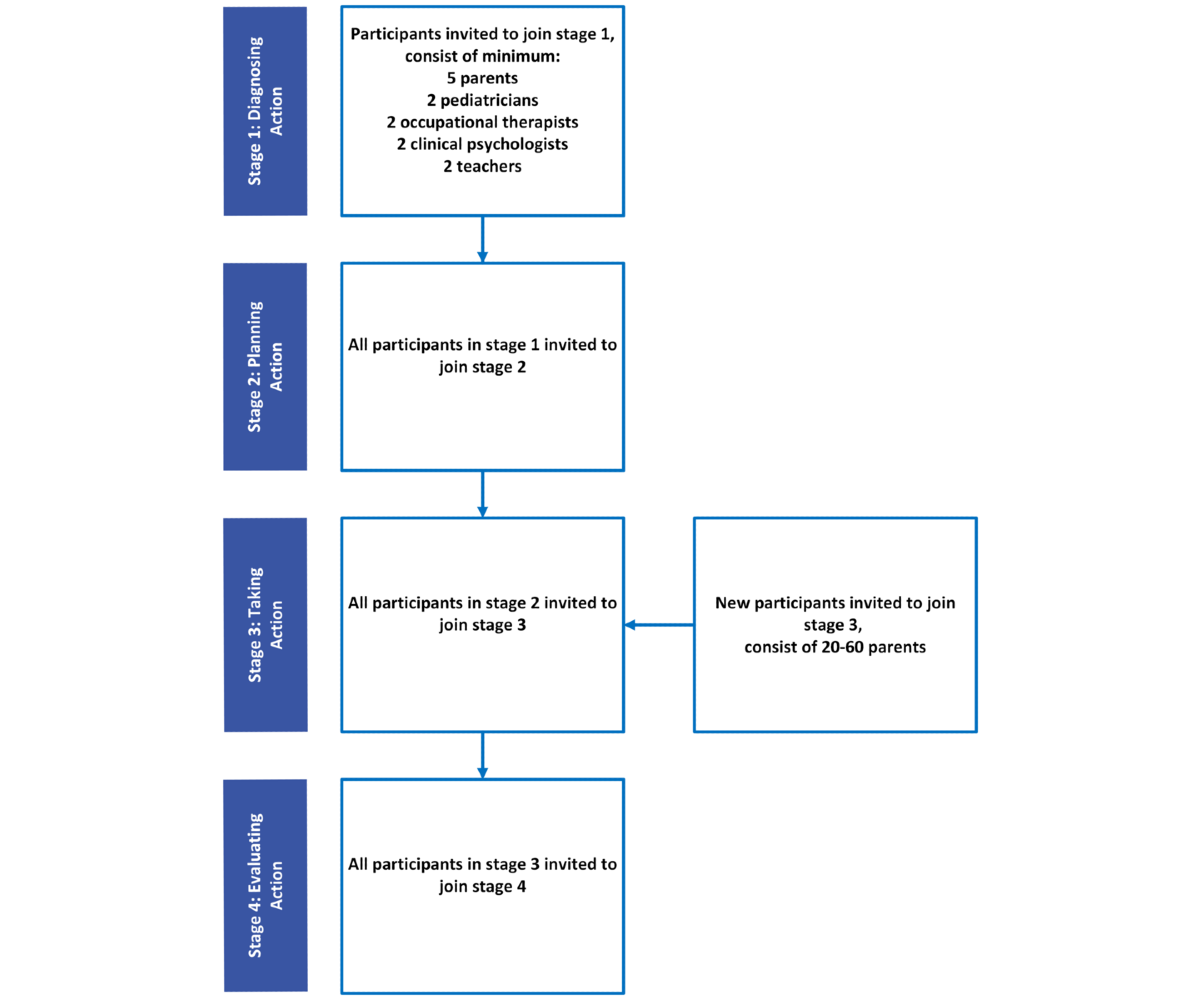
Participant flow diagram for the action research stages.

The evaluation in stage 4 will involve both quantitative and qualitative assessments of the mobile-based PHR. Participants in stage 4 will consist of those who have participated from stage 1 to stage 3, as well as additional participants newly recruited in stage 3. Qualitative evaluations will be conducted exclusively with participants who have been involved from stage 1 to stage 3, as they can provide comprehensive and holistic feedback based on their entire experience. Meanwhile, quantitative evaluations in stage 4 will focus solely on parent participants, including both parents who participated from stage 1 to stage 3 and newly recruited parents in stage 3. The primary reason for focusing the quantitative assessment on parents is that they will be the main users of the mobile-based PHR, which is being designed to support the management and care of their children with ADHD. Figure 2 illustrates the sequential involvement of participants, starting from the initial recruitment in stage 1 through their continued engagement up to stage 4.

#### Data Collection

Quantitative data to assess the usefulness of the mobile-based PHR will be collected using the Indonesian version of the System Usability Scale (SUS) questionnaire [69]. The SUS consists of 10 statements, each scored on a Likert scale of 1-5, where a higher score indicates stronger agreement with the statement [70]. Additionally, quantitative data will be gathered to assess the quality of the mobile-based PHR using the User Version of the Mobile App Rating Scale (uMARS) questionnaire. The uMARS includes 20 items divided into 5 dimensions: engagement, functionality, aesthetics, information, and subjective quality. Each statement is scored on a 5-point Likert scale [71]. Qualitative data will also be collected through in-depth interviews and FGDs to gather comprehensive insights from participants’ experiences during stage 3 (taking action). This approach will aim to uncover rich, contextual information regarding usability, satisfaction, challenges encountered, and participants’ input and expectations for further development of the mobile-based PHR.

#### Data Analysis

Quantitative data obtained from the SUS questionnaire will be analyzed descriptively. The results will be summarized, totaled, and then multiplied by a coefficient of 2.5, yielding a final score within a range of 0-100 [70]. This score will be used to determine the percentile ranking. Data from the uMARS questionnaire will be analyzed through descriptive statistical analysis, calculating the mean (SD) to assess the quality of the mobile app. Scores ranging from 1 to 2 indicate “not acceptable,” 2 to 3 indicate “poor,” 3 to 4 indicate “acceptable,” 4 to 5 indicate “good,” and 5 indicate “excellent” quality [72]. Qualitative data from in-depth interviews and FGDs will be analyzed using thematic analysis [49].

### Trustworthiness

The data in this research will be validated using the action research validation technique, which comprises coherence validation, discursive validation, and practical validation [35]. Coherence validation focuses on how various empirical, interpretative, and conceptual elements are integrated to mutually reinforce each other. The coherence validation technique that will be used in this research is triangulation, a method that will involve cross-referencing the results from FGDs and in-depth interviews regarding user requirements with the findings from the reviews of paper-based PHRs, particularly related to data that must be recorded in the PHR.

Discursive validation emphasizes dialogical and democratic validation in a forum. The discursive validation technique that will be used in this research is member checking, a method that involves returning the research findings to the participants for their review and validation. This iterative process will allow participants to confirm the accuracy and relevance of the data collected during in-depth interviews, FGDs, document reviews, and other research activities. By incorporating member checking, the study aims to ensure the credibility and trustworthiness of the research outcomes, as participants can provide feedback and insights, contributing to the overall validation and refinement of the research findings.

Practical validation focuses on testing in a real environment so that specific knowledge claims can gain sufficient trustworthiness. The practical validation method that will be used in this research is participant engagement, providing participants with an opportunity to actively use the mobile app prototype. Unlike a passive observation approach, participants will be directly involved in navigating and interacting with the prototype, offering insights into the user experience from a firsthand perspective. This hands-on engagement will ensure a more practical and realistic assessment of the mobile app’s functionality and usability. By allowing participants to explore the prototype independently, the study aims to gather authentic feedback on the user interface, features, and overall performance, contributing to the refinement and enhancement of the mobile app based on the practical experiences of the end users.

The summary of the action research activities in each stage including output, activities, instrument, variable, participants, and validation methods are presented in Multimedia Appendix 1.

### Ethical Considerations

The study has been approved by the Ethics Committee of the Faculty of Medicine, Public Health, and Nursing, Universitas Gadjah Mada (approval no: KE-FK-1257-EC-2023). To maintain ethical standards, we will provide comprehensive information regarding the study protocol to all potential participants. We will outline the purpose, procedure, and potential risks and benefits of their participation, especially in the context of action research [73]. Once they agree to participate, they will be required to provide signed informed consent. Confidentiality and privacy will be rigorously conducted throughout the research process, with measures in place to anonymize and secure sensitive information [74].

## Results

This study was initiated in October 2024, with the research process divided into 4 distinct stages:

- Stage 1 (diagnosing action) will focus on identifying user requirements and challenges in managing ADHD care through stakeholder interviews, FGDs, and document reviews. As of December 2024, 13 participants were enrolled, comprising 5 (38.5%) parents, 2 (15.4%) pediatricians, 2 (15.4%) occupational therapists, 2 (15.4%) clinical psychologists, and 2 (15.4%) teachers, meeting the minimum target for participant numbers. This stage was completed at the end of 2024, providing key insights to guide subsequent stages.
- Stage 2 (planning action) will include a systematic literature review, user requirement validation, mockup design validation, and prototype development. The prototype development will be conducted in collaboration with a technical development team. This stage is projected to conclude by September 2025, delivering a finalized prototype for usability testing.
- Stage 3 (taking action) will focus on implementing and testing the prototype with participants during a 6-week trial period. During this phase, system usage data, user feedback, and error reports will be collected and analyzed to refine the prototype. This stage is anticipated to be completed by the end of 2025.
- Stage 4 (evaluation action) will involve both qualitative and quantitative assessments of the mobile-based PHR. Qualitative evaluations will gather in-depth feedback from participants who have engaged in all stages, providing comprehensive insights into the user experience. Quantitative evaluations, targeting all parent participants, will assess the usability and quality of the mobile app through embedded questionnaires. This stage is expected to conclude in February 2026. The results of this study will be reported according to reporting guidelines provided for action research [75]. The final results are anticipated for publication by the middle of 2026.

## Discussion

### Summary

The landscape of future research in pediatric ADHD stands to evolve significantly, particularly in the development of effective electronic systems tailored for gathering crucial information to diagnose and monitor children and adolescents with ADHD [76]. As an innovative response to this research avenue, this study will contribute to this domain by using an action research approach that engages the stakeholders in pediatric ADHD management in all stages, including diagnosing, planning, taking, and evaluating action [77]. To the best of our knowledge, this is the first study that involves active participation and engagement with stakeholders, including parents, medical providers, and nonmedical providers, in the development of a mobile app related to pediatric ADHD management. This dynamic methodology will ensure that the mobile app developed is technologically effective, user-friendly, and contextually relevant.

The insights gleaned from this research will underscore the intricate challenges associated with managing pediatric ADHD and emphasize the critical role of collaborative and patient-centric interventions. These principal findings will pave the way for more targeted and contextually relevant interventions, fostering a deeper understanding of the intricacies involved in pediatric ADHD management.

### Limitations

The scope of the study is confined to addressing the needs of stakeholders directly involved in the routine care of children with ADHD within tertiary hospital settings. Although this focus provides valuable insights into the specific challenges faced in such contexts, the findings may not be fully representative of the broader spectrum of ADHD management, especially in community or primary care settings. Additionally, the study’s outcomes may be influenced by the regional or institutional characteristics of the selected tertiary hospitals, potentially limiting the generalizability of the results to a more diverse health care landscape. Despite these limitations, the study remains dedicated to refining and optimizing the care of children with ADHD within tertiary hospital environments, with the anticipation that the insights gained can contribute meaningfully to the broader discourse on pediatric ADHD management.

### Conclusion

This study protocol outlines a pivotal initiative to enhance the management of ADHD in pediatric populations. By using an action research methodology and actively engaging stakeholders, including parents, medical providers, and nonmedical providers, the study aims to contribute significantly to the field. The iterative cycles of the research seek to develop a mobile-based PHR that is not only user-friendly but also effective and uniquely attuned to the diverse needs of those involved in pediatric ADHD care.

## Appendix

Multimedia Appendix 1 Summary of action research activities.

## Abbreviations

ADHD: attention-deficit/hyperactivity disorder
FGD: focus group discussion
MeSH: Medical Subject Headings
PHR: personal health record
SUS: System Usability Scale
uMARS: User Version of the Mobile App Rating Scale

## Data Availability

The datasets used and analyzed during this study will be made available from the corresponding author upon reasonable request after the completion of the study.
