# Supplementary material for: Development of a Mobile-Based Personal Health Record for Pediatric Attention-Deficit/Hyperactivity Disorder Management: Protocol for a Study Based on Action Research Design
Source: JMIR Res Protoc. 2025 Apr 10;14:e60216. doi: 10.2196/60216 (PMC12022525; doi:10.2196/60216)
Supplement: Multimedia Appendix 1 [file resprot_v14i1e60216_app1.pdf]

The summary of the action research activities

Stages	Output	Activities	Instrument	Variable	Participants	Validation
**Diagnosing action**						
Identify user requirements	Specific requirements for the development of a mobile-based PHR	Document review	Review form	User requirements	Paper-based PHR	Coherence validation: triangulation, Discursive validation: member checking
		In-depth interviews and FGD	Interview and FGD guideline, Audio recording	User requirements	parents, pediatricians, occupational therapists, clinical psychologists, and teachers	
**Planning action**						
Design and develop mobile-based PHR prototype	Agreed design and prototype	Systematic literature review	Systematic review protocol	User requirements	Principal investigators	Discursive validation: member checking
		Consensus	Consensus form, smartphone, app installer	Participants agreement	parents, pediatricians, occupational therapists, clinical psychologists, and teachers	Discursive validation: member checking Practical validation: Prototype trial
**Taking action**						
Test the mobile-based PHR	User-reported error logbook	User recruitment and training	User manual, smartphone, app installer	User-reported errors	parents, pediatricians, occupational therapists, clinical psychologists, and teachers	Practical validation: Prototype testing

Stages	Output	Activities	Instrument	Variable	Participants	Validation
**Evaluating action**						
Evaluate the mobile-based PHR	Usability assessment result	Questionnaires distribution	Questionnaires form (SUS)	Mobile app usefulness	parents, pediatricians, occupational therapists, clinical psychologists, and teachers	Practical validation: Prototype testing
	Quality assessment result	Questionnaires distribution	Questionnaires form (uMARS)	Mobile app quality	parents, pediatricians, occupational therapists, clinical psychologists, and teachers	Practical validation: Prototype testing
	User experiences	In-depth interviews and FGD	Interview and FGD guideline, Audio recording	User input and expectation for further development	parents, pediatricians, occupational therapists, clinical psychologists, and teachers	Discursive validation: member checking
